# Supplementary material for: Hybrid Models and Biological Model Reduction with PyDSTool
Source: PLoS Comput Biol. 2012 Aug 9;8(8):e1002628. doi: 10.1371/journal.pcbi.1002628 (PMC3415397; doi:10.1371/journal.pcbi.1002628)
Supplement: Text S4 — Complete source code for the PyDSTool package (version 0.88.120504). Includes API documentation and help files linking to web pages. This file is identical to the current public release on Sourceforge.net. (ZIP) [file pcbi.1002628.s004.zip › PyDSTool/html/toc-PyDSTool.Toolbox.optimizers.criterion.criteria-module.html]

xml version="1.0" encoding="ascii"?


criteria


# Module criteria

---

## Classes

AbsoluteParametersCriterion

AbsoluteValueCriterion

GradientCriterion

IterationCriterion

MonotonyCriterion

RelativeParametersCriterion

RelativeValueCriterion

---

[hide private]
